# Adaptive selection of quasispecies during *in vivo* passaging in chickens, mice, and ferrets results in host-specific strains for the H9N2 avian influenza virus

**DOI:** 10.1128/jvi.00151-25

**Published:** 2025-05-08

**Authors:** Yiliang Li, Xi Quan, Rujian Chen, Xiao Wang, Yiting Chen, Yingde Gan, David M. Irwin, Yongyi Shen

**Affiliations:** 1 Guangdong Laboratory for Lingnan Modern Agriculture, State Key Laboratory for Animal Disease Control and Prevention, Center for Emerging and Zoonotic Diseases, College of Veterinary Medicine, South China Agricultural University 554665, Guangzhou, China; 2 Department of Laboratory Medicine and Pathobiology, University of Toronto 233837, Toronto, Ontario, Canada; 3 Banting and Best Diabetes Centre, University of Toronto 7938, Toronto, Ontario, Canada; 4 School of Agriculture and Biology, Shanghai Jiao Tong University 117750, Shanghai, China; 5 Guangdong Provincial Key Laboratory of Zoonosis Prevention and Control, Guangzhou, China; Cornell University Baker Institute for Animal Health, Ithaca, New York, USA

**Keywords:** receptor-biding preference, H9N2, avian influenza virus, random mutation

## Abstract

Sporadic human infections of avian influenza virus (AIV) raise significant public health concerns. A critical factor limiting the transmission of AIVs is the shift in receptor-binding preference from Siaα2,3 to Siaα2,6. To reveal the adaptive selection dynamics during the host adaptation process of AIVs, this study generated a viral library with random mutations in the HA gene of the H9N2 strain. Upon passaging the viral library in chickens and mice, the predominantly selected variants exhibited a preference for Siaα2,3 receptors. Notably, the wild-type strain remained dominant in both inoculated and direct-contact chickens, while variants with the ΔL226/R229I substitutions were preferentially selected in mice. Ferrets have a predominance of Siaα2,6 in their respiratory tract. As expected, the variant harboring the N289D mutation, which prefers Siaα2,6 binding, was enriched during *in vivo* passaging in ferrets. The mice-adapted variant with the ΔL226/R229I mutations causes reduced levels of TNF-α in the early days post-infection in mice, which correlated with an increase in its viral titers. Conversely, elevated levels of IL-6 and IL-1β at five dpi may contribute to the development of the cytokine release syndrome, potentially elucidating the higher fatality rate observed. In conclusion, based on the mutant spectra of the HA gene, this study elucidates the distinct quasispecies dynamics during the adaptation of H9N2 to different hosts, with receptor availability serving as one of the driving factors. Furthermore, a series of critical substitutions that influence the interspecific transmission potential of H9N2 AIVs were identified.

**IMPORTANCE:**

The mutation of viruses creates a quasispecies reservoir. In this study, we aimed to investigate the dynamics of quasispecies during the host adaptation of AIVs. We generated a viral library with random mutations in the HA gene of H9N2 and conducted serial passaging in chickens, mice, and ferrets for five generations, respectively. The wild-type strain was dominant in chickens, while mice selected viruses with the ΔL226/R229I substitutions. Both variants showed a preference for binding to Siaα2,3, which aligned with the abundance of Siaα2,3 found in the respiratory tract epithelial cells of chickens and mice. In ferrets, where Siaα2,6 is more prevalent, the variant with the N289D mutation, which prefers Siaα2,6, was found to be enriched. In summary, this study revealed the adaptive selection of H9N2 quasispecies in various hosts, contributing to our understanding of AIV host adaptation.

## INTRODUCTION

Influenza A viruses (IAVs) are negative-sense, single-stranded RNA viruses. To date, all subtypes of IAVs, with the exception of H17N10 and H18N11—which are exclusively isolated from bats—can be found in avian (1). Avian influenza A viruses (AIVs), particularly the H5, H7, and H9 subtypes, have caused great economic losses to the poultry industry (2–4). The ongoing circulation and mutation of these viruses in birds result in host adaptation of AIVs to humans, thereby posing a substantial threat to public health (5, 6). The H9N2 subtype characterized as a low pathogenic AIV represents a prominent subtype of AIVs since its discovery in 1994 (7). This subtype continues to evolve, resulting in increased genetic diversity and the emergence of antigenically novel strains. Recent studies have indicated that certain avian-origin H9N2 viruses exhibit a strong affinity for human-type viral receptors (8–10). Sporadic infections in mammals, including humans, have been reported (11–13). Furthermore, serological surveillance has revealed that between 2.3 and 13.7% of poultry-exposed workers were positive for anti-H9 antibodies (14). These observations have raised significant concerns regarding the potential for this virus to contribute to a future pandemic.

RNA viruses are characterized by high mutation rates during replication, which results in great genetic diversity. This stochastic nature of mutations allows a viral population, referred to as quasispecies, to rapidly adapt to fluctuating environmental conditions (15, 16). From the perspective of quasispecies and general genetics, the mutant spectra can function as units of selection (17). The presence of a diverse array of mutant genomes within a viral population provides a reservoir of both genotypic and phenotypic variants, thereby enhancing the viruses’ capacity for adaptability in response to changing environments (18, 19). During the process of human adaptation of AIVs, adaptive evolution signals were detected, and most adaptive amino acids in the HA gene were proved to correlate with the functional adaptation from avian to human (20, 21).

AIVs generally demonstrate a preference for binding to sialic acid α2,3 (Siaα2,3) receptors, while viruses of humans exhibit a greater affinity for Siaα2,6 receptors (22, 23). The adaptive change in receptor specificity from Siaα2,3 to Siaα2,6 receptors is considered to be a prerequisite for the AIVs to infect humans and cause pandemics (24–26). Hemagglutinin (HA), a vital surface glycoprotein of AIVs, plays a significant role in this process. Certain substitutions within the HA sequence can alter the receptor specificity of AIVs from Siaα2,3 to Siaα2,6 receptors (10, 27–30). Notably, specific amino acid substitutions in the HA protein, such as Q226L and G228S, have been extensively documented to be associated with the adaptation of AIVs to human-type receptors (25, 31, 32).

Given the significant role of the HA gene of AIVs in determining receptor-binding preferences, in this study, we randomly mutated the H9N2 HA gene to generate a random mutant spectrum of viral quasispecies. These quasispecies were subsequently serially passaged *in vivo* in chickens, mice, and ferrets for five generations to elucidate the dynamics of quasispecies adaptation to novel host environments.

## RESULTS

### Overview for random mutational scanning for the HA gene

**Fig 1:**
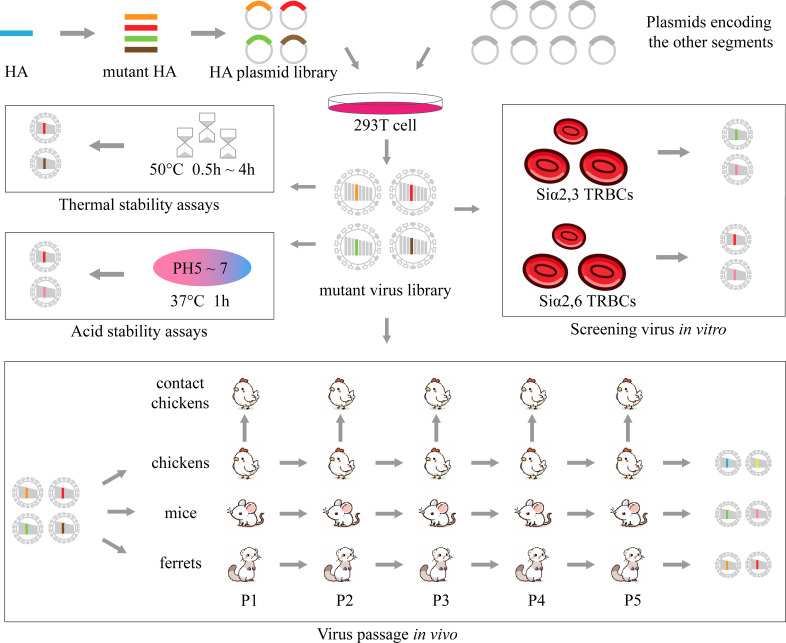
Overview of the experimental design to reveal substitutions in the HA gene that determine the adaptation of the H9N2 virus. Random mutations were introduced into the entire HA gene of the wild-type HY strain. The rescued mutant viral library was *in vitro* incubated at 50°C for 0.5, 1, 2, 3, and 4 h to identify mutations associated with thermal tolerance and incubated in pH-adjusted PBS solutions (pH levels ranging from 5 to 7) for 1 h at 37°C to identify mutations associated with acid tolerance. Furthermore, the viral library was serially passaged *in vivo* in chickens, mice, and ferrets for five passages (P1 to P5) to select host-adapted variants.

In order to generate random mutant spectra of viral quasispecies for the H9N2 virus and evaluate their adaptive selection in different environments, we used the approach outlined in Fig. 1. First, the HA gene of the wild-type H9N2 strain (designated as HY) was randomly mutagenized and used to rescue a mutant viral library, which exhibited an average of 8.7 nucleotide mutations per variant sequence in the HA gene (Fig. S1A). The average numbers of nonsense, synonymous, and missense mutations per variant were 0.2, 2.7, and 5.8, respectively, accounting for 2.4, 30.7, and 66.9% of the total mutations, respectively (Fig. S1B). The mutant viral library was then rescued and screened through both *in vitro* and *in vivo* experiments to select adaptive variants for avian- or human-type receptor preferences. The experimental procedures included: (i) incubation of the mutant viral library with modified TRBCs that were modified to predominantly express Siaα2,6 or Siaα2,3 on their cell surface, aimed at selecting virus strains with a preference for binding to either Siaα2,6 or Siaα2,3, respectively; (ii) assessment of thermal tolerance by incubating the library at 50°C for 0.5, 1, 2, 3, and 4 h and characterizing the surviving viruses; (iii) evaluation of acid tolerance through incubation of the library was incubated in a series of PBS solutions adjusted to pH levels ranging from 5 to 7 for 1 h at 37°C, followed by characterization of the surviving viruses; and (iv) serial passage of the library *in vivo* in three species—chickens, mice, and ferrets, for five generations to assess adaptation to these hosts.

### Incubation of the viral library *in vitro* with siaα2,6-TRBCs and Siaα2,3-TRBCs

TRBCs have both Siaα2,6 and Siaα2,3 on their cell surface (Fig. S2A). After sialic acids were removed (Fig. S2B), Siaα2,6 or Siaα2,3 was reintroduced to the de-sialylated TRBCs to create Siaα2,6-TRBCs and Siaα2,3-TRBCs, respectively (Fig. S2C and D).

**Fig 2:**
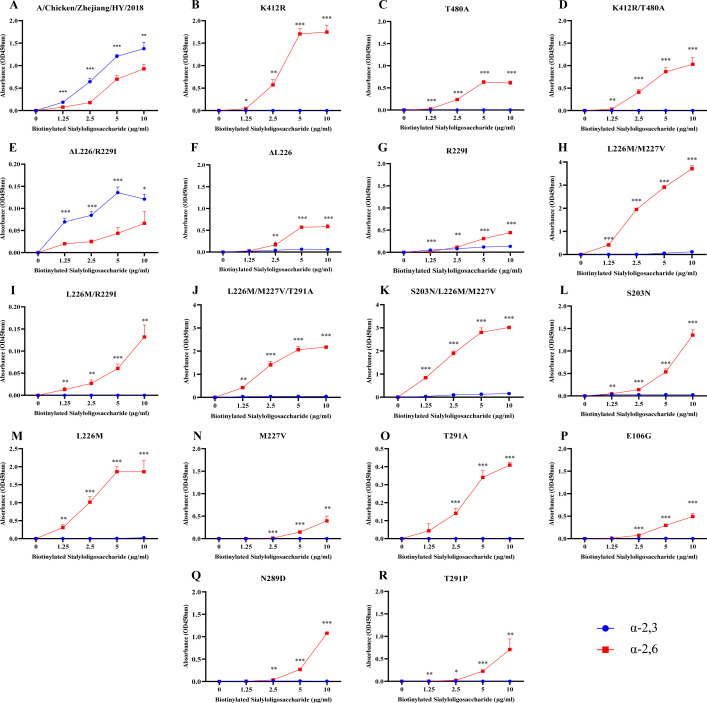
Receptor-binding properties of H9N2 viruses with different HA amino acid substitutions. (A) HY, (B) K412R, (C) T480A, (D) K412R/T480A; (E) ΔL226/R229I, (F) ΔL226, (G) R229I, (H) L226M/M227V, (I) L226M/R229I, (J) L226M/M227V/T291A, (K) S203N/L226M/M227V, (L) S203N, (M) L226M, (N) M227V, (O) T291A, (P) E106G, (Q) N289D, and (R) T291P. The data shown represent the mean values from three independent replicates, with the aerror bars denoting the standard deviations. The receptor binding preference of these viral variants was determined using a range of concentrations of sialic acids conjugated to biotinylated sialylglycopolymers (3′SLN and 6′SLN) via direct solid-phase binding assays. Statistical analyses were performed using a *t*-test. *, **, and *** indicate *P* values of less than 0.05, 0.01, and 0.001, respectively.

The viral library was incubated with Siaα2,6-TRBCs or Siaα2,3-TRBCs at 4°C, followed by extensive washing to remove nonspecific or weakly bound viruses. PacBio long-read sequencing revealed that the variant with the simultaneous presence of K412R and T480A substitutions constituted 75.6% of the viral variants after filtering by Siaα2,6-TRBCs. Accordingly, 94.7 and 94.6% of the variants exhibited the K412R and T480A substitutions in the HA gene, respectively, whereas only 3.5 and 2.5% of variants displayed these substitutions when screened with the Siaα2,3-TRBCs (Table S1). Receptor-binding assays showed that, in comparison to the wild-type HY strain, which prefers Siaα2,3 (Fig. 2A), variants with K412R, T480A, and K412R/T480A substitutions exhibited preference for Siaα2,6 (Fig. 2B through D).

Additionally, the co-mutation of ΔL226 (deletion of L at position 226) and R229I in HA was observed in 73.5% of the variants after filtering with the Siaα2,3-TRBCs. Accordingly, 90.4 and 94.2% of total variants have these two mutations, respectively, in contrast to only 2.9 and 1.0% observed with the Siaα2,6-TRBC treatment. Receptor-binding experiments showed that variants with the co-occurrence of the ΔL226 and R229I in the HA gene favored binding to Siaα2,3, while variants possessing either ΔL226 or R229I alone favored binding to Siaα2,6 (Fig. 2E through G).

### Mutations associated with thermal and acid tolerance

The environmental factors of ambient temperature and pH significantly impact the viability and persistence of the virus in the environment (33–35), thereby impacting their transmissibility. Consequently, this study aimed to identify mutations that confer tolerance to elevated temperatures and acidic conditions.

**Fig 3:**
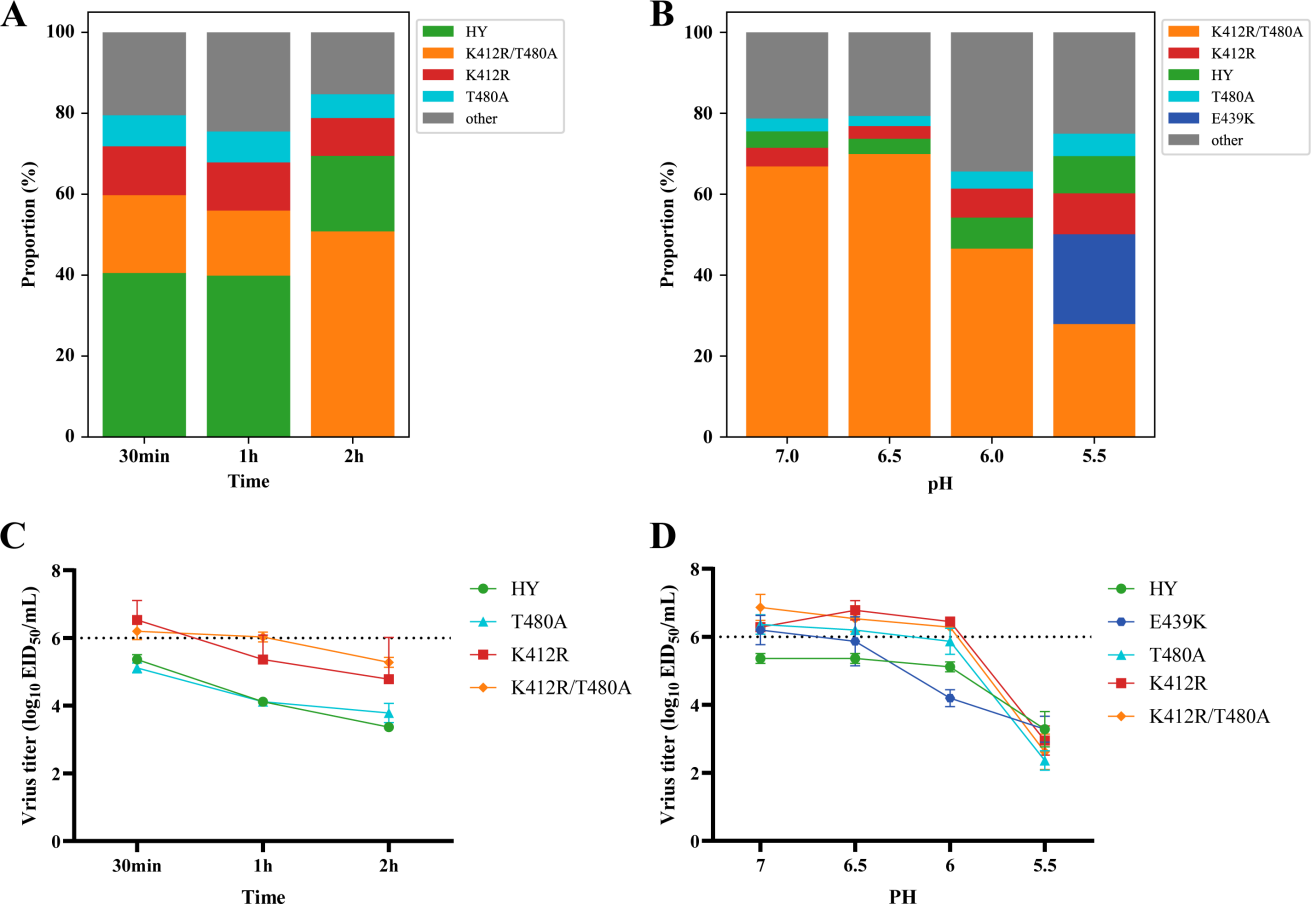
Thermal and acid tolerance of viral variants. (A) The profiles of viral variants after thermal treatment. The viral library was incubated at 50°C for 0.5, 1, 2, 3, and 4 h. Subsequently, the viral solutions were propagated in specific-pathogen-free (SPF) 10-day-old embryonated chicken eggs. Notably, treatments lasting 3 and 4 h did not yield viable viruses; thus, only the data corresponding to 0.5, 1, and 2 h are displayed. (B) Profiles of viral variants after treatments with acid. The viral library was incubated for 1 h at 37°C in PBS buffer with pH levels adjusted between 5.0 and 7.0 (specifically, 5.0, 5.5, 6.0, 6.5, and 7.0). Following these treatments, the virus solutions were then propagated in SPF embryonated chicken eggs. Viable viruses were not recovered at pH 5.0. Therefore, only data for pH levels of 5.5, 6.0, 6.5, and 7.0 are shown. The composition of the viral library prior to any treatment is detailed in Fig. S1. (C) Thermal tolerance of the wild-type HY, HY/HA-T480A, HY/HA-K412R, and HY/HA-K412R/T480A strains. Each strain (10^6^ EID50) was incubated in a metal bath at 50°C for 0.5, 1, 2, 3, or 4 h prior to conducting the EID_50_ assay. No viable viruses were recovered after 3 or 4 h of exposure; thus, only data from the 0.5, 1, and 2 h were analyzed. (D) Acid tolerance of HY, HY/HA-T480A, HY/HA-K412R, HY/HA-K412R/T480A, and HY/HA-E439K strains. Each strain (10^6^ EID_50_) was incubated in buffers at pH 7.0, 6.5, 6.0, and 5.5 for 1 h at 37°C before the EID_50_ assay was conducted. The dashed line indicates the input virus titer of all strains. Data are expressed as mean ± standard deviation from three independent experiments.

To identify substitutions associated with the tolerance of high temperature, the viral library was incubated at 50°C for 0.5, 1, 2, 3, and 4 h. No live viruses were recovered after 3 or 4 h of exposure; thus, only data from the 0.5, 1, and 2 h were analyzed. At the 0.5 and 1 h treatments, the wild-type HY strain predominated, constituting 40.5 and 39.9% of the population, respectively. However, after 2 h of treatment, the proportion of the HY strain decreased to 18.7%, while the variant harboring the K412R/T480A substitution increased from 16.1 to 50.1%. The K412R and T480A variants were also detected in the 0.5, 1, and 2 h treatments, with their proportions ranging from 9.3 to 12.1% and 5.9 to 7.7%, respectively (Fig. 3A).

To identify substitutions associated with acid tolerance, the mutant viral library was incubated in PBS solutions adjusted to pH levels ranging from 5.0 to 7.0. No viruses survived treatment in a buffer at a pH of 5.0; thus, only data from pH levels of 5.5 to 7.0 were shown. The variant HY/HA-E439K was the most prevalent at pH 5.5, comprising 22.2% of the population. The HY/HA-K412R and HY/HA-T480A variants were present at proportions of 3.1 to 10.1% and 2.5 to 5.6%, respectively, across pH levels from 5.5 to 7.0. Notably, the variant with the K412R/T480A substitutions exhibited the highest abundance, ranging from 46.6 to 69.9% at pH levels of 6.0, 6.5, and 7.0. The wild-type HY represented 3.8 to 9.2% of the population at pH levels between 5.5 and 7.0 (Fig. 3B).

Based on these results, viruses with the E439K, T480A, K412R, and K412R/T480A substitutions, which may influence temperature or acid tolerance, were selected for further testing. In thermal tolerance assays, the viral titers of the HY/HA-K412R and HY/HA-K412R/T480A variants were significantly higher than those of the wild-type HY strain at all time points (*P* < 0.01), whereas the T480A substitution alone did not enhance thermotolerance (Fig. 3C). In the acid tolerance assays, viral titers for all viruses decreased 10-fold following exposure to the pH 5.5 buffer. Viral titers for the HY/HA-K412R and HY/HA-K412R/T480A variants had the best acid tolerance, exhibiting a loss of less than 1 log_10_ in infectivity after exposure to buffers at pH levels of 6.0 to 7.0, which was 1–2 log_10_ higher than that observed for any other strain (Fig. 3D).

### Differential preferences for viral variants during serial passaging in chickens, mice, and ferrets

The mutant viral library was serially passaged in chickens, mice, and ferrets for five generations. At the end of the passaging experiment, the adapted viral strains exhibited distinct variations among these species (Fig. 5). In inoculated and direct-contact chickens, the only variant that exceeded a 5% prevalence was the wild-type HY strain, which maintained average proportions ranging from 75.6 to 77.0% across the five passages.

**Fig 4:**
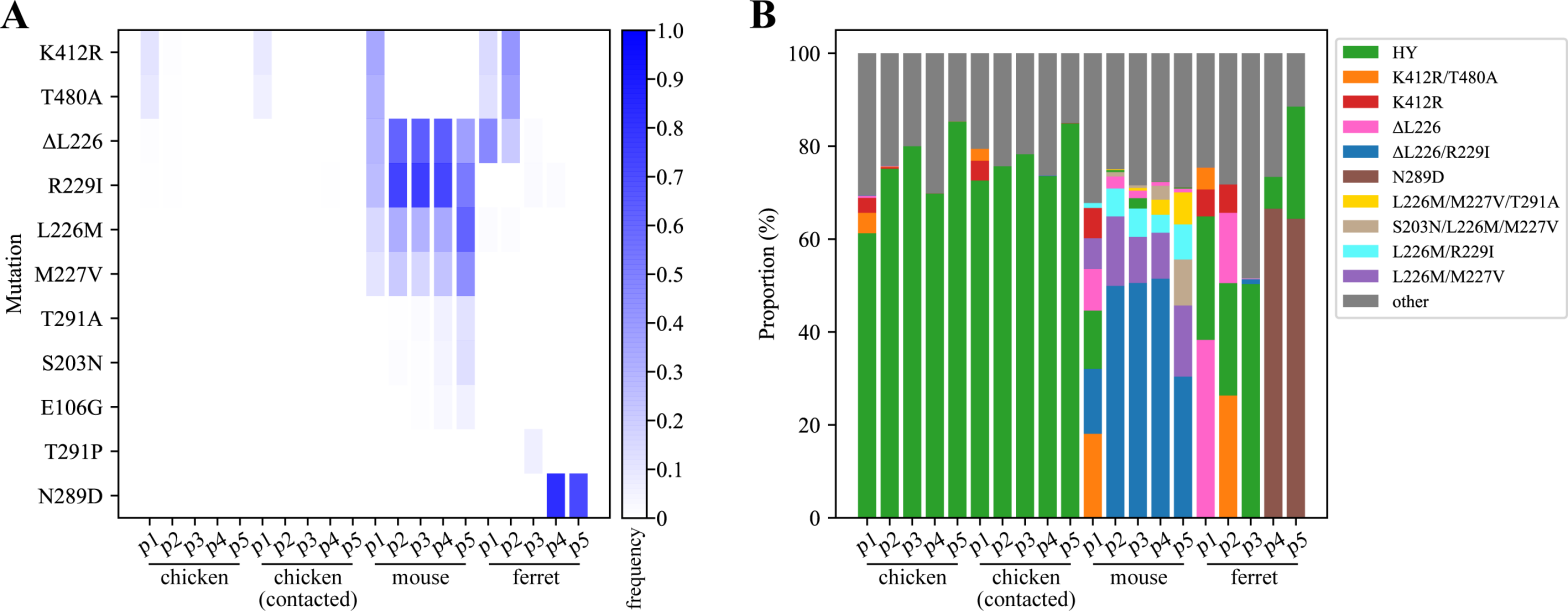
Overview of the *in vivo* animal passaging experiments. (A) Heat map of the distribution of HA substitution variants across various passages in different species. The vertical axis displays variants that exhibit a frequency exceeding 5% in any sample, while the horizontal axis represents the animal samples. P1–P5 represent the passage generation. (B) Stacked histogram shows the predominant viral variants in each sample during the passaging process. The horizontal axis represents animal samples and passage numbers, whereas the vertical axis indicates the proportion of the various variants identified. The different colors denote distinct variants, with the category labeled “other” indicating variants that constitute less than 5% in any sample. The composition of the viral library prior to any treatment is detailed in Fig. S1.

**Fig 5:**
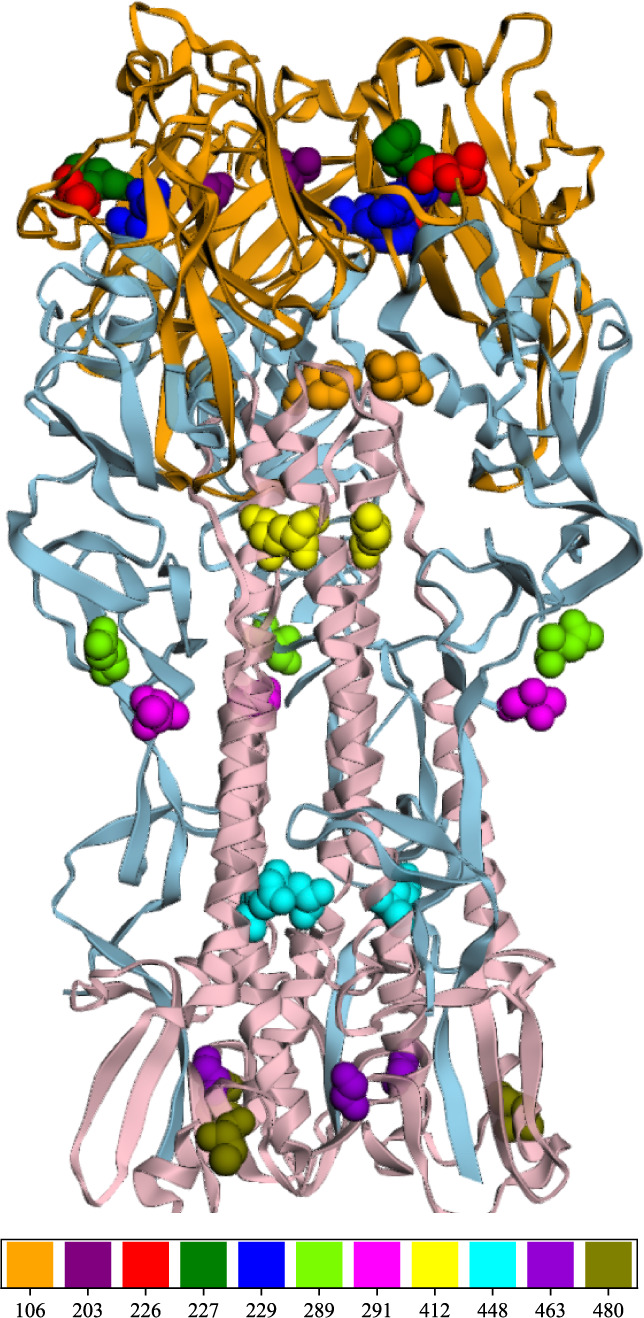
Positions of amino acid mutations on a three-dimensional structural model of the HA protein derived from the H9N2 virus (PDB: 1JSH). The ribbon diagram is color-coded according to subdomains: the receptor subdomain is represented in orange, the vestigial enzyme subdomain in blue, and the HA2 stem F subdomain in pink.

In mice, the wild-type HY, along with several variants, including HY/HA-K412R/T480A, HY/HA-K412R, HY/HA-ΔL226, HY/HA-ΔL226/R229I, HY/HA-L226M/M227V/T291A, HY/HA-S203N/L226M/M227V, HY/HA-L226M/R229I, and HY/HA-L226M/M227V, all had proportions exceeding 5% in at least one passage. The wild-type HY strain exhibited a proportion of 12.5% in passage 1, which subsequently declined to below 5% in passages 2 through 5. The variants HY/HA-K412R/T480A and HY/HA-K412R, both of which preferentially bind to Siaα2,6 (Fig. 2B and D), accounted for 18.1 and 6.5% of the population, respectively, after passage 1, but their prevalence diminished to below 5% in subsequent passages. The HY/HA-ΔL226 variant, also favoring Siaα2,6 (Fig. 2F), had a proportion of 8.9% in passage 1, with subsequent proportions falling below 5% in passages 2 through 5. Conversely, the HY/HA-ΔL226/R229I variant, which preferentially binds to Siα2,3 (Fig. 2E), represented 14.0% of the population after passage 1 and subsequently increased in prevalence, becoming the dominant haplotype in passages 2 through 5, with proportions ranging from 30.4 to51.5%. The HY/HA-L226M/M227V variant, which prefers Siaα2,6 (Fig. 2H), exhibited proportions between 6.6 and 15.3% across passages 1 to 5. The HY/HA-L226M/R229I variant was identified in all passages but only showed frequencies exceeding 5% in passages 2, 3, and 5, with proportions of 6.0, 6.1, and 7.6%, respectively. This variant preferentially binds to Siaα2,6 (Fig. 2I). The variants HY/HA-L226M/M227V/T291A and HY/HA-S203N/L226M/M227V, which also favor Siα2,6 (Fig. 2J and K), were first detected in passage 2 and exhibited higher proportions of 6.7 and 9.9%, respectively, in passage 5. To further examine whether these site combinations must function in concert or if certain sites are merely enriched as a result of a hitchhiking effect, we additionally rescued strains harboring a single mutation site and assessed their receptor binding preferences. The variants harboring individual substitutions (S203N, L226M, M227V, R229I, T291A) preferentially bind to Siα2,6 (Fig. 2G and L through O). The frequency of the HY/HA-E106G variant exceeded 5% only in passage 5 in mice (5.3%), (Fig. 4A, not shown in Fig. 4B due to its unique appearance) and has a preference for Siaα2,6 (Fig. 2P). The enrich mutation, following the passaging of the mutant library in mice, predominantly occurs within the receptor-binding domain (Fig. 5). Of particular significance are the K412R and T480A, which are located in the C-terminal.

In ferrets, the wild-type HY strain was detected in passages 1 through 5, exhibiting proportions that varied from 6.8 to 50.3%. The HY/HA-ΔL226 variant, with a preference for Siaα2,6, was detected in passages 1 and 2, with 38.3 and 15.1% proportions of the population, respectively. The HY/HA-K412R variant, which prefers Siaα2,6, was detected in passages 1 and 2, with 5.8 and 6.1% proportions, respectively. Additionally, the HY/HA-K412R/T480A variant, with a preference for Siaα2,6, was detected in passages 1 and 2, with proportions of 4.7 and 26.3%, respectively. The HY/HA-N289D variant emerged as the dominant strain in passages 4 and 5, accounting for 66.6 and 64.4% of the population, respectively. This strain prefers Siaα2,6 (Fig. 2Q). Furthermore, the HY/HA-T291P variant was detected in passage 3, with a frequency of 6.3% (Fig. 4A), and this substitution also results in a preference for Siaα2,6 for the virus (Fig. 2R).

### HY/HA-ΔL226/R229I variant is highly pathogenic in mice

**Fig 6:**
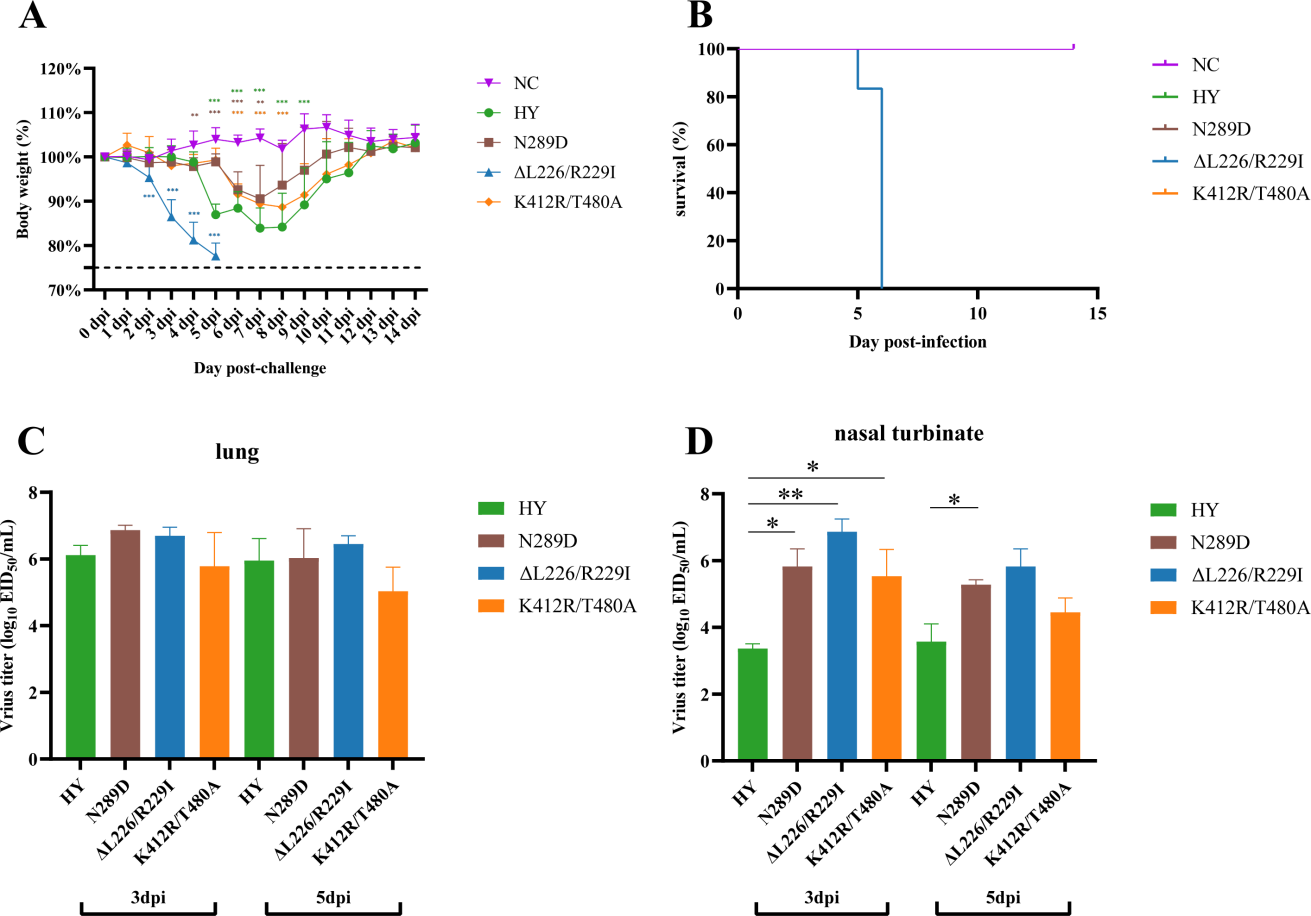
Pathogenicity of the wild-type HY, HY/HA-N289D, HY/HA-ΔL226/R229I, and HY/HA-K412R/T480A viral strains in mice. (A) Changes in body weight and (B) mortality rates in mice. Viral titers from the lung tissues (C) and nasal turbinate tissues (D) of the mice at 3 and 5 dpi. Six-week-old female C57BL/6 mice (*n* = 12 per group) were intranasally inoculated with 50 µL of 10^6^ EID_50_/mL HA variant virus and monitored daily for changes in body weight and mortality daily for 14 days. The mean viral titers from three mice are shown, with error bars representing standard deviations.

The HY/HA-ΔL226/R229I and the wild-type HY strains were identified as the predominant strains after serial passaging in mice and chickens, respectively. The HY/HA-K412R/T480A variant emerged as the dominant strain after thermal tolerance treatments, while the HY/HA-N289D variant was the major strain identified in ferrets. The pathogenicity of these variants was further assessed in mice. Following the challenge with these viral strains, mice were monitored for weight changes for 2 weeks. All groups of mice that underwent viral challenges experienced significant weight loss, with those infected by the HY/HA-ΔL226/R229I strain exhibiting a trend toward the most rapid weight loss (Fig. 6A). Notably, all mice inoculated with the HY/HA-ΔL226/R229I strain died within 6 dpi, whereas mice inoculated with the other strains survived throughout the observation period (Fig. 6B). Virus titer was similar in the lung tissue for all four viral groups (Fig. 6C). However, the viral titer in the nasal turbinate of mice inoculated with the wild-type HY strain was found to be 1–4 log units lower than that of the other groups at both 3 and 5 dpi (Fig. 6D). Mice inoculated with the HY/HA-ΔL226/R229I variant exhibited the highest viral titers in the nasal turbinate at both 3 and 5 dpi.

**Fig 7:**
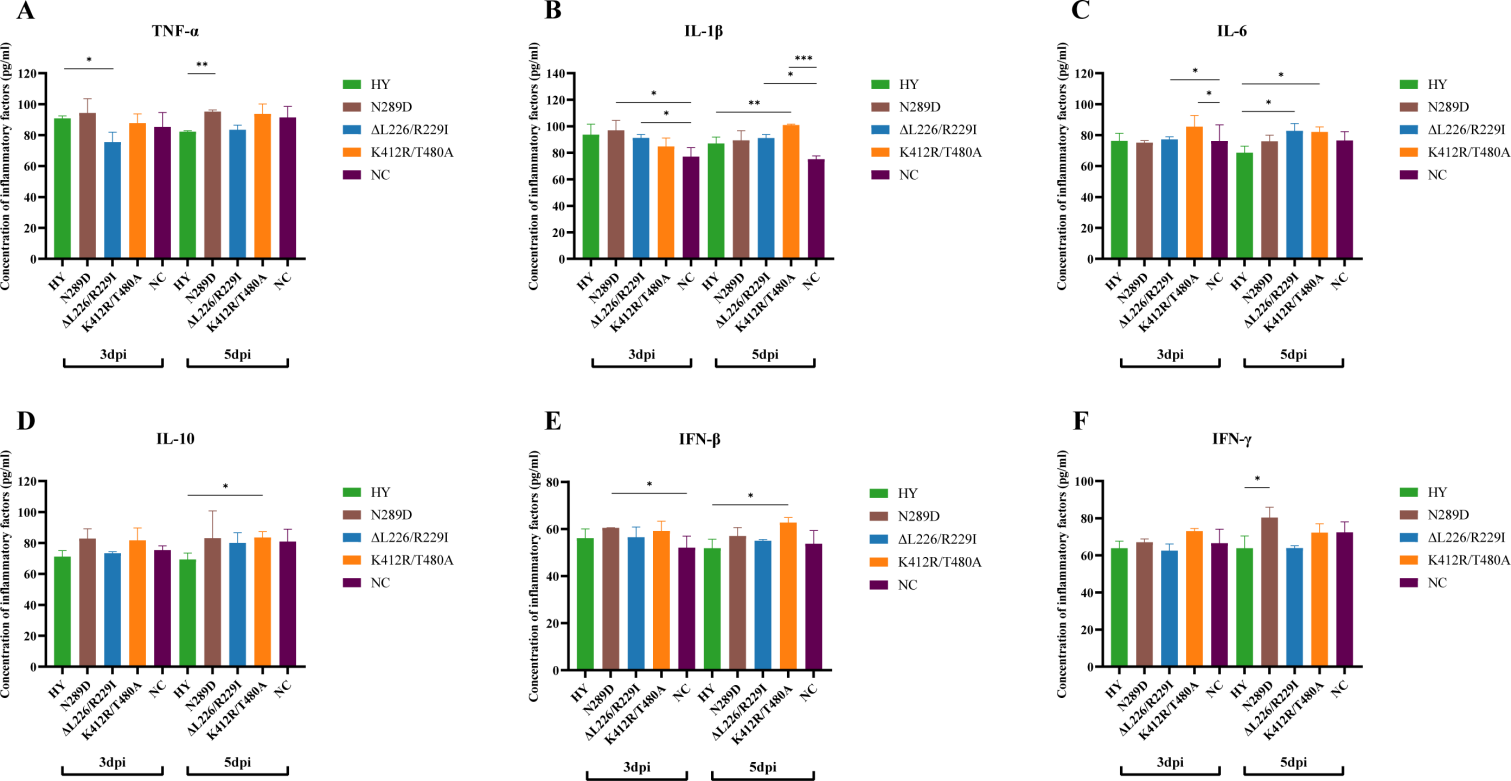
Levels of cytokines after the challenge with the wild-type HY, HY/HA-N289D, HY/HA-ΔL226/R229I, and HY/HA-K412R/T480A viral strains in mice. Six-week-old female C57BL/6 mice (*n* = 12 per group) were intranasally inoculated with 50 µL of 10^6^ EID_50_/mL of the HA variant virus. Serum samples were collected at 3 and 5 dpi. (A) TNF-α, (B) IL-1β, (C) IL-6, (D) IL-10, (E) IFN-β, and (F) IFN-γ. Statistical analyses were performed using a *t*-test. *, **, and *** indicate *P* values of less than 0.05, 0.01, and 0.001, respectively.

After the viral challenge, the levels of TNF-α, IL-1β, IL-6, IL-10, IFN-β, and IFN-γ were found to be similar among the four viral strains at 3 dpi, with the exception of the HY/HA-ΔL226/R229I variant, which had significantly lower TNF-α levels than in the HY-infected mouse (*P* < 0.05, Fig. 7). At 5 dpi, levels of IL-6 and IL-1β were significantly higher in mice infected with the HY/HA-ΔL226/R229I strain compared to those infected with the HY strain and the negative control (NC) group. Additionally, TNF-α and IFN-γ levels were significantly higher in the HY/HA-N289D-infected mice relative to the HY-infected group. Furthermore, IL-6, IL-10, IL-1β, and IFN-β levels were significantly increased in mice infected with the HY/HA-K412R/T480A variant compared to those infected with the HY strain.

**Fig 8:**
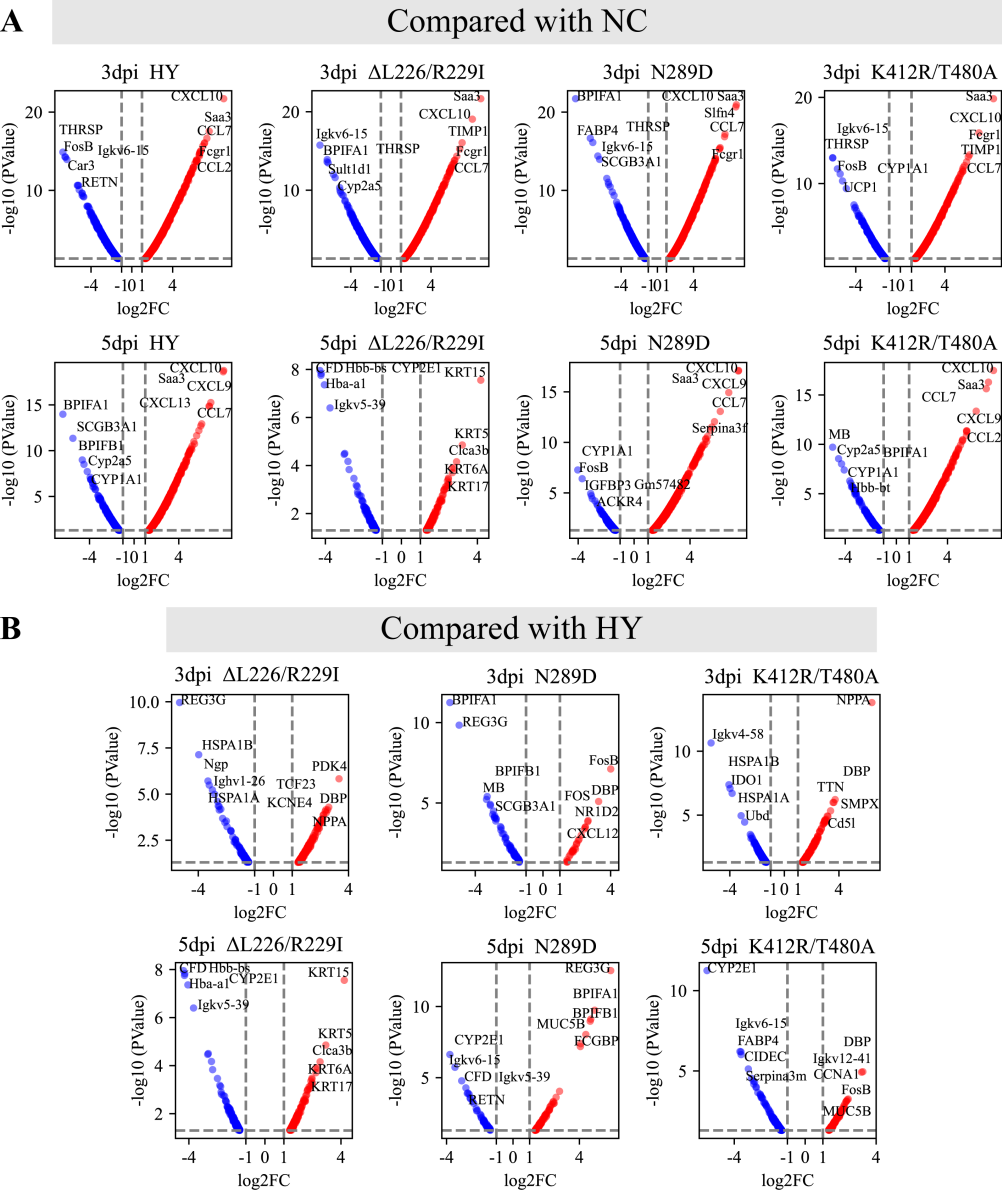
Volcano plot of differentially expressed genes. The *x*-axis represents the log2 scale of the fold change in gene expression (log2(Fold Change)). Negative values indicate downregulation, and positive values indicate upregulation. The *y*-axis displays the negative log10 scale of the *P* value (–log10(*P* value)), which indicates the significance level of the observed expression differences. Red dots represent significantly upregulated genes, while blue dots represent significantly downregulated genes. Genes that do not exhibit significant differential are not shown in the graph. The most differentially expressed genes are labeled in the plot. (A) Four viral challenge groups compared with the control (NC) groups. (B) Three mutant viral groups (HY/HA-K412R/T480A, HY/HA-N289D, and HY/HA-ΔL226/R229I) compared with the wild-type HY group.

We used RNA-seq to analyze the gene expression profiles of the lung tissue from mice in all of the viral challenge groups and the NC group at 3 and 5 dpi. In comparison to the NC group, a total of 4,017 differentially expressed genes (DEGs) (*P* ≤ 0.05 and log2FC ≥ 1) were identified. The four viral challenge groups exhibited similar compositions of their upregulated DEGs (Fig. 8A; Fig. S3A). Notably, all viral strains resulted in the upregulation of the *Cxcl9*, *Cxcl10*, *Saa3*, *Fcgr1*, and *Ccl7* genes. Compared to the wild-type HY strain, 916 DEGs were identified in other groups. The DEGs identified among the three mutant strains are very different, with few overlapping genes (Fig. 8B; Fig. S3B).

**Fig 9:**
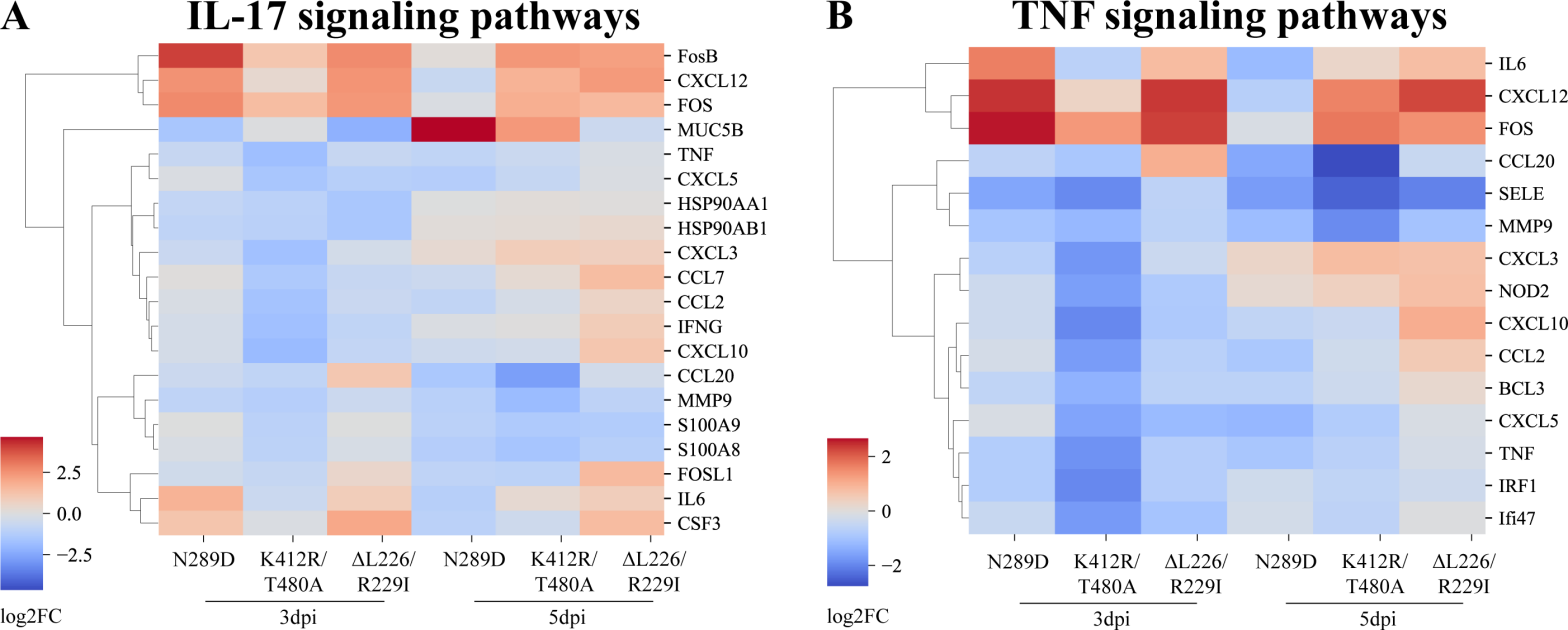
Expression profile of genes involved in IL-17 (A) and TNF (B) signaling pathways. Each grid cell represents a log2 fold change (log2FC) of the selected differentially expressed genes (DEGs) in the specified HA mutant viral strain compared to the wild-type HY strain at the designated time points. The *x*-axis labels indicate the various mutant viral strains at the different days post-infection (dpi). The *y*-axis lists the DEGs involved in the respective IL-17 (A) or TNF (B) signaling pathways. The color gradient represents the log2FC values, with red indicating upregulation and blue indicating downregulation of the corresponding gene in the mutant relative to the wild-type strain. The clustering of rows (genes) and columns (samples) is performed based on the similarities in their expression patterns.

As shown in Fig. S4, Gene Ontology (GO) and Kyoto Encyclopedia of Genes and Genomes (KEGG) analyses of the DEGs revealed that, compared to the HY strain, genes upregulated after the HY/HA-ΔL226/R229I challenge at 5 dpi were predominantly enriched in immune-related categories and pathways, including “leukocyte chemotaxis,” “IL-17 signaling pathway,” and “cytokine-cytokine receptor interaction.” It is noteworthy that the “IL-17 signaling pathway” was downregulated at 3 dpi. The downregulated genes were enriched in categories, such as “immunoglobulin production” and “production of molecular mediator of immune response.” In the case of mice infected with the HY/HA-N289D strain, upregulated genes were enriched in the “IL-17 signaling pathway” at 3 dpi, while downregulated genes were enriched in the “viral protein interaction with cytokine receptor” pathway at 5 dpi. The immune response in mice infected with the HY/HA-K412R/T480A strain exhibited greater complexity. At 3 dpi, downregulated genes were enriched in terms related to virus, such as “response to virus,” “cellular response to interferon-beta,” “response to interferon-gamma,” “TNF signaling pathway,” and “viral protein interaction with cytokine and cytokine receptor.” At 5 dpi, the “IL-17 signaling pathway” was simultaneously enriched in both upregulated and downregulated genes according to the KEGG pathway analysis. Additionally, downregulated genes were enriched in pathways related to viral and interferon responses. The DEGs related to the immune response in mice infected with the mutant strains were primarily enriched in pathways associated with the IL-17 and TNF signals. We clustered the relative expression levels of these DEGs (20 genes for the IL-17 signaling pathway and 15 for the TNF signaling pathway), revealing that the relative expression levels of these immune pathway-related DEGs were significantly higher in the HY/HA-ΔL226/R229I-infected mice at 5 dpi compared to the other viral mutants (Fig. 9).

## DISCUSSION

Public health is continually threatened by AIVs. H9N2 has become a dominant AIV subtype in poultry. Moreover, H9N2 infections in mammals such as pigs, minks, horses, canines, and humans have been sporadically documented, highlighting its potential threat to public health and its potential to cause a further pandemic (11–13). Therefore, a comprehensive understanding of the evolutionary dynamics of the virus during its adaptation to new hosts is essential.

The significant difference in receptor preference between human and avian influenza viruses is considered to be one of the most important factors contributing to species-specific barriers. The HA receptor-binding preference for Siaα2,6 (human-type receptors) is a crucial initial step in the zoonotic transmission of AIVs to humans (36). In this study, a random mutant viral library was screened using Siaα2,6-TRBCs and Siaα2,3-TRBCs to identify amino acid substitutions in the HA protein that correlate with receptor preference. The substitutions K412R, T480A, and K412R/T480A in HA were found to confer a preference for Siaα2,6 in the viruses. These strains emerged as the predominant variants following selection with Siaα2,6-TRBCs (Fig. 2B through D). In contrast, the co-mutation of ΔL226 and R229I in HA was observed in 73.5% of the variants identified using Siaα2,3-TRBCs. Viral passaging of the random mutant viral library in mice also favored the HY/HA-ΔL226/R229I variant as the dominant strain (Fig. 5). Given that Siaα2,3 receptors are prevalent in the respiratory epithelial cells of mice (37), this observation supports the conclusion that the dominant variant, HY/HA-ΔL226/R229I, exhibits a preference for Siaα2,3, with its frequency increasing in correlation with the number of passages. To examine whether the multiple substitutions function cooperatively or are simply due to hitchhiking, viruses with single HA substitutions, R229I or ΔL226, were also reconstructed. Variants exhibiting individual HA substitutions resulted in a preference for Siaα2,6, indicating that the presence of either ΔL226 or R229I in HA shifts receptor preference toward Siaα2,6. Conversely, the variant with dual substitutions, ΔL226/R229I, maintains a preference for Siaα2,3. Thus, our findings suggest that an epistatic interaction between these substitutions in HA may influence the phenotypic outcome. Previous studies exploring the molecular mechanisms of host adaptation of AIVs have primarily focused on individual substitutions; thus, our results highlight the importance of considering the interconnectedness of mutations in influencing receptor preference.

In chickens, although both Siaα2,3 and Siaα2,6 receptors are present throughout the respiratory tract and gut, Siaα2,3 is expressed at significantly higher levels (37). During the passage of the mutational viral library in chickens, the predominant haplotype in both infected and direct-contact chickens was the wild-type HY strain, which prefers Siaα2,3. This suggests that the HY strain is already well-adapted to the chickens. Both chickens and mice have a higher abundance of Siaα2,3. As expected, the dominant strains in these species show a preference for Siaα2,3. Nevertheless, their predominant haplotypes were very different, with wild-type HY strain being prevalent in chickens and HY/HA-ΔL226/R229I in mice. This finding suggests that, in addition to receptor binding, other factors may also play a critical role in the host adaptation of AIVs. In a prior report, it was demonstrated that the R229I mutation enhances the fitness of drug-resistant strains of the H7N9 virus in mammalian cells by augmenting the binding affinity of the virus to Siaα2,3 and Siaα2,6 receptors (38). In the present study, we observed that this mutation facilitates the H9N2 virus in developing a preference for the Siaα2,6 receptor.

In comparison to mice and chickens, the distribution of sialic acid receptors in ferrets and humans exhibits greater similarity characterized by a predominance of Siaα2,6 receptors and a lesser presence of Siaα2,3 receptors on the epithelial cells of the respiratory tract. This receptor distribution underpins the extensive utilization of ferrets as a model for investigating human influenza viruses (39, 40). Unlike the screen with Siaα2,6-TRBCs, which resulted in the identification of the predominant variant HY/HA-K412R/T480A, the primary variant identified in ferrets was HY/HA-N289D, which demonstrates a preference for binding to Siaα2,6.

The HY/HA-K412R/T480A variant exhibits significantly enhanced thermal tolerance and acid tolerance. Such tolerance is critical for viral dissemination, as it enables the virus to endure prolonged exposure to adverse environmental conditions, thereby enhancing its transmission potential (33–35). This variant demonstrates a preference for binding to Siaα2,6. Three secondary structural elements of the HA protein—the 130-loop, the 190-helix, and the 220-loop—constitute the boundaries of the receptor-binding site (41). Notably, the aforementioned mutations do not reside within the receptor-binding domain (Fig. 5), necessitating further research to elucidate their role in modifying the receptor binding preference of this strain. The HY/HA-K412R/T480A variant’s capacity to bind to human receptors, coupled with its superior environmental adaptability (improved thermal and acid tolerance), underscores its heightened public health risk.

In this study, compared to HY/HA-N289D, HY/HA-K412R/T480A, and wild-type HY strains, the mice-adapted HY/HA-ΔL226/R229I strain exhibited a significantly higher pathogenicity in mice, achieving a mortality rate of 100%. Furthermore, the replication capacity of this strain in the respiratory tract of mice was markedly superior to that of the wild-type HY strain (Fig. 5D). After infection, the expression levels of *CXCL10*, *Saa3*, and *CCL7* were found to be elevated across all viral-infected groups (Fig. 8A; Fig. S3A). These three genes encode the chemokine CXCL10 (recruitment of T and NK cells), the acute phase protein Saa3 (marker of inflammatory response), and the chemokine CCL7 (recruitment of monocytes/macrophages), thereby contributing to the antiviral immune response (42, 43). Dysregulation of immune function often results in the excessive production of inflammatory cytokines and chemokines, leading to respiratory inflammation (44). Therefore, we measured the levels of inflammatory cytokines in the serum of infected mice. After infection of mice with the HY/HA-ΔL226/R229I strain, levels of TNF-α decreased at 3 dpi, while IL-6 and IL-1β levels increased at 5 dpi (Fig. 7). TNF-α is known to facilitate the infiltration of macrophages, dendritic cells, NK cells, and neutrophils into affected tissues to attempt to control and clear infections (45). The decline in TNF-α levels in the early days of infection by the HY/HA-ΔL226/R229I strain correlates with an increase in viral titers (Fig. 6D). IL-6 is involved in the development of cytokine release syndrome, with elevated levels of IL-6 and IL-1β being closely associated with poor prognoses in influenza patients and animal models (46, 47). The increases in IL-6 and IL-1β levels at 5 dpi are consistent with the high mortality rate associated with infection by the HY/HA-ΔL226/R229I strain. Gene expression analyses further support the hypothesis that this virus provokes a severe immune response during the later stages of infection (5 dpi). The HY/HA-ΔL226/R229I strain was found to upregulate a larger number of DEGs in IL-17 and TNF signaling pathways compared to other strains (Fig. 9). This finding suggests that infection with HY/HA-ΔL226/R229I induced the recruitment of a substantial number of immune cells, activating multiple cytokine signaling cascades, which may culminate in a cytokine storm (48). Simultaneously, genes that were downregulated by this virus were enriched in categories related to “immunoglobulin production” and “production of molecular mediator of immune response” (Fig. S4), indicating a potential evasion of host immune clearance through the suppression of antibody production and immune mediator secretion (49, 50). The excessive immune response triggered by this mutant strain may be a critical factor contributing to the observed mortality in these infected mice.

H9N2 AIVs are widely distributed globally and have the potential to cross species barriers from poultry to human. In this study, based on random mutant spectra, we revealed their quasispecies dynamics and identified a series of novel sites associated with receptor preference during their adaptation to new hosts.

## MATERIALS AND METHODS

### Generation of a viral library incorporating random mutations in the HA gene

The wild-type HY strain of H9N2 AIV used in this study was isolated from sick chickens in Zhejiang Province, China in 2018 (GenBank accession numbers OK035258 to OK035265). Random mutations were introduced into the HA gene using a PCR Random Mutagenesis Kit (Clontech, Mountain View, CA, USA). Subsequently, the resulting random mutation HA plasmid library was used to generate a viral library in the genetic background of the seven additional plasmids that encoded the remaining viral genes (NA, NP, M, NS, PB1, PB2, and PA) through reverse genetics (51). Briefly, eight plasmids (0.6 µg of each plasmid) were co-transfected into 293T cells. After 24 h, the medium was replaced with 2 mL of reduced serum medium (Opti-MEM) with 1 mg/mL TPCK-Trypsin and 10 mg/mL bovine serum albumin. At 72 h post-transfection, the culture supernatant was harvested and subsequently inoculated into specific pathogen-free (SPF) 10-day-old embryonated chicken eggs to produce viral stocks.

### Screening virus variants that prefer siaα2,6 or Siaα2,3 receptors

To select virus variants within the viral library that exhibit the capacity to bind to Siaα2,6 or Siaα2,3 receptors, we prepared turkey red blood cells (TRBCs) that predominantly possess Siaα2,6 or Siaα2,3 on their cell surfaces as previously described (52). Briefly, 100 µL of 10% TRBCs was incubated with 50 mU of *Vibrio cholerae* neuraminidase (Sigma, St. Louis, MO, USA) at 37°C for 20 min to remove sialic acids from the cell surfaces of TRBCs. Subsequently, the de-sialylated TRBCs were incubated with either 2 mU of α2–6(N)-sialyltransferase (Sigma, St. Louis, MO, USA) to generate Siaα2,6-TRBCs or 2.5 mU of α2–3(N)-sialyltransferase (Sigma, St. Louis, MO, USA) in conjunction with 1.5 mM cytidine-5′-monophospho-N-acetylneuramine acid (Sigma, St. Louis, MO, USA) to generate Siaα2,3-TRBCs for 1 h at 37°C. After cleaning the TRBCs three times using phosphate-buffered saline (PBS), the re-sialylated TRBCs were suspended in PBS. Finally, the re-sialylated TRBCs were incubated with 1 µL of FITC-SNA (Vector Laboratories, Burlingame, CA, USA) or 1 µL of FITC-MAL II (Vector Laboratories, Burlingame, CA, USA) for 5 min at 25°C. Subsequently, the re-sialylated TRBCs were analyzed via flow cytometry to ascertain the successful re-sialylation of the TRBCs.

To select HA virus variants capable of binding to Siaα2,6 or Siaα2,3, the viral library was incubated with 0.1 mL of 10% TRBCs that predominantly possess Siaα2,6 (Siaα2,6-TRBCs) or Siaα2,3 (Siaα2,3-TRBCs) on the cell surface for 10 min at 4°C, and then centrifuged at 2,000 RPM for 2 min. The sediment was then washed 10 times using minimum essential medium (Gibco, Grand Island, NY, USA) containing 313 mM NaCl. Subsequently, the washed TRBCs were incubated at 37°C for 30 min to obtain the bound viruses. The experiment was conducted in triplicate. The sediment from the three independent experiments was mixed together to extract total RNA. The HA gene was then amplified by RT-PCR and sent for third-generation sequencing.

### *In vivo* selection for adapted H9N2 HA mutant viruses in mice, chickens, and ferrets

Six-week-old SPF chickens, 6-week-old C57BL/6J mice, and 4–6-month-old ferrets that were serologically negative for influenza virus were used for the following experiments. For each generation, three individuals from each species were used. Chickens, mice, and ferrets in the initial generation were intranasally inoculated with 200, 50, and 500 µL of 10^6^ EID_50_ of the H9N2 viral library, respectively. For the chicken experiment, three chickens were designated as the direct-contact group. At 3 (for chickens and mice) or 4 (for ferrets) days post-inoculation (or post-exposure), animals were euthanized, and nasal turbinate (for chickens and ferrets) or lung tissues (for mice) were harvested. These tissues were subsequently homogenized, with the supernatants utilized for inoculation of the subsequent generation of animals. The HA gene was also amplified from the supernatants and sent for third-generation sequencing. A total of five generations were serially passaged to facilitate the selection of adapted viruses in these animals.

### Acid and thermal tolerance of HA protein variants

We followed a procedure to assess the acid tolerance of the viruses *in vitro*, as previously described (53). The PBS (Gibco, Grand Island, NY, USA) was adjusted to the target pH levels of 7.0, 6.5, 6.0, 5.5, and 5.0 by using 0.1 M citric acid. The viruses were diluted in PBS and incubated at 37°C for 1 h. The thermotolerance test was performed as described in a previous study (52). Specifically, viruses (10^6^ EID_50_/mL units in PBS) were transferred to 1.5 mL tubes and incubated in a metal bath at 50°C for 0.5, 1, 2, 3, or 4 h. After incubation, the virus solutions were propagated in 10-day-old SPF embryonated chicken eggs to eliminate inactive strains. After that, HA genes were amplified and sent for third-generation sequencing. It is important to note that these treatments may have resulted in the emergence of certain egg-adaptive mutations. Consequently, following the filtration process, we proceeded to rescue the adaptive strains and repeated the pH and temperature treatments.

### Third-generation sequencing and data analysis

Total RNA was extracted from the HA mutant viral library, as well as from the nasal turbinate or lung tissues of the experimental animals. The HA genes were then amplified and used for library preparation and sequencing on the PacBio Sequel platform. An average of 49,309 sequencing reads per sample was obtained.

The raw sequences were initially processed via the PacBio SMRT portal. Sequences were filtered to ensure a minimum of three passes and a minimum predicted accuracy of 90% (minfullpass = 3, minPredictedAccuacy = 0.9). This threshold of 90% predicted accuracy is established to differentiate between high-quality circular consensus sequences and noise. The reads were assigned to their respective samples based on unique barcodes, and the barcode and primer sequences were subsequently truncated.

The clean reads were mapped to the reference sequence (HA gene of the wild-type HY strain) by minimap2 (version = 2.26) with the parameters “-x map-hifi and—cs=short.” Further mutation analysis was performed using a custom Python (version = 3.9.16) script developed in the JupyterLab (version = 3.6.3) environment. The region selected for mutational analysis encompassed residues 19–498 of the influenza HA protein, as delineated by the three-dimensional structure of H9N2 (54).

### Site-directed mutagenesis

Mutations were introduced into the HA gene using fusion PCR. In order to investigate whether the predominant variants, which possess multiple mutations, exhibit cooperative effects or if the observed substitutions are merely a result of hitchhiking, viruses with individual mutations were also rescued. Primers are listed in Table S2. Subsequently, the mutagenized HA genes were used to rescue viruses by reverse genetics (Table S3).

### Receptor-binding assay

The H9-specific monoclonal antibody (Mab) 2G10 was kindly provided by Z. Wan (Yangzhou University, China). The receptor-binding properties of recombinant H9N2 viruses were analyzed using a solid-phase binding assay, as previously described (55). Briefly, 96-well plates coated with high binding capacity streptavidin (Nunc, Carlsbad, CA, USA) were sequentially coated with twofold dilutions of Neu5Aca2-3Galb1-4GlcNAcb-PAA-biotin and Neu5Aca2-6Galb1-4GlcNAcb-PAA-biotin (Glycitech, USA) and incubated overnight at 4°C. After blocking with 5% skim milk in phosphate-buffered saline with Tween 20 (PBST), the plates were incubated overnight at 4°C with 64 hemagglutination units of the H9N2 viruses. After three washes with PBST, the plates were incubated with H9-specific MAb 2G10 at 4°C for 2 h. The plates were then washed three times with PBST and incubated with HRP-labeled Goat Anti-Mouse IgG (H + L) (Biyuntian, Nanjing, China) for 2 h at 4°C, followed by three washes with PBST. Next, 100 µL of TMB solution (Solarbio, Beijing, China) was added to each well and incubated for 10 min. The reaction was terminated by adding 50 µL of enzyme-linked immunosorbent assay (ELISA) Stop Solution (Solarbio, Beijing, China), and the OD450 values were then measured.

### Viral challenge experiments in mice

Viral strains exhibiting the K412R/T480A, ΔL226/R229I, and N289D substitutions in HA, along with wild-type HY strains, were selected for mouse challenge experiments. For each viral strain, a total of 12 6-week-old female C57BL/6 mice (Laboratory Animal Center, Southern Medical University) were intranasally inoculated with 50  µL of 10^6^ EID_50_ of the virus. Mice in the control group were inoculated with PBS instead. Mice in all groups were monitored daily for 14 days to assess weight loss and survival rates. Mice that lost exceeding 25% of their initial body weight were humanely euthanized, and this was recorded as a mortality event. For each group, three mice were randomly chosen and euthanized at 3 and 5 dpi. Their nasal turbinate and lung tissues and serum were collected and stored at −80°C. A part of the nasal turbinate and lung tissues was homogenized and used for viral titration, while additional lung samples were used for transcriptome sequencing and analysis. Serum samples were collected from the infected mice selected for euthanization at 3 and 5 dpi, and the levels of IL-1β, IL-10, IL-6, TNF-α, IFN-γ, and IFN-β in the serum were measured using ELISA assay kits (Meimian, China) following the manufacturers’ instructions.

### Transcriptome sequencing and analysis

Total RNA was extracted from the lung tissues of both infected and negative control mice using TRIzol (Invitrogen, San Diego, CA, USA) according to the manufacturer’s instructions. RNA-Seq was conducted on the Illumina Hiseq NovaSeq6000 sequencer (Illumina, San Diego, CA, USA). After filtering low-quality reads and trimming adapter sequences, trimmed clean data were mapped to the mouse reference genome (GRCm38) using Hisat2 (v2.2.1) (56). Gene expression levels were quantified using featureCounts (v2.0.6) (57). Differential gene expression analysis was performed using edgeR (v3.32.1) (58), with genes exhibiting |logFC| > 1 classified as differentially expressed genes. Python (v3.9.16) was used to visualize the DEGs. GO and KEGG analyses were conducted using the R software (v.4.2.2) package clusterProfiler (v.4.5.0) (59) via Hiplot Pro (https://hiplot.com.cn/), a comprehensive web-based platform for biomedical data analysis and visualization.

## Data Availability

All high-throughput sequencing data were deposited to the NCBI SRA database under BioProject accession no. PRJNA1110385. The code utilized for sequencing analyses can be accessed at the following URL: https://github.com/lvlvlvyulv/h9n2_rand_mut/.

## SUPPLEMENTAL MATERIAL

Figure S1 jvi.00151-25-s0001.tif Overview of the random mutations in the HA gene of the of the mutant viral library.

Figure S2 jvi.00151-25-s0002.tif Binding affinity of MAL II or SNA to turkey red blood cells assessed through flow cytometry analysis.

Figure S3 jvi.00151-25-s0003.tif Link diagrams connecting the most differentially expressed genes to samples collected at various time points and from different mutant viral strains.

Figure S4 jvi.00151-25-s0004.tif Bidirectional bar chart displaying the results of an enrichment analysis for DEGs in mice infected with various mutant HA viral strains at 3 and 5 dpi.

Supplemental material jvi.00151-25-s0005.docx Tables S1 to S3; legends for Fig. S1 to S4.

ASM does not own the copyrights to Supplemental Material that may be linked to, or accessed through, an article. The authors have granted ASM a non-exclusive, world-wide license to publish the Supplemental Material files. Please contact the corresponding author directly for reuse.
