# Supplementary figures and images for: Adaptive selection of quasispecies during in vivo passaging in chickens, mice, and ferrets results in host-specific strains for the H9N2 avian influenza virus
Source: J Virol. 2025 May 8;99(6):e00151-25. doi: 10.1128/jvi.00151-25 (PMC12172485; doi:10.1128/jvi.00151-25)

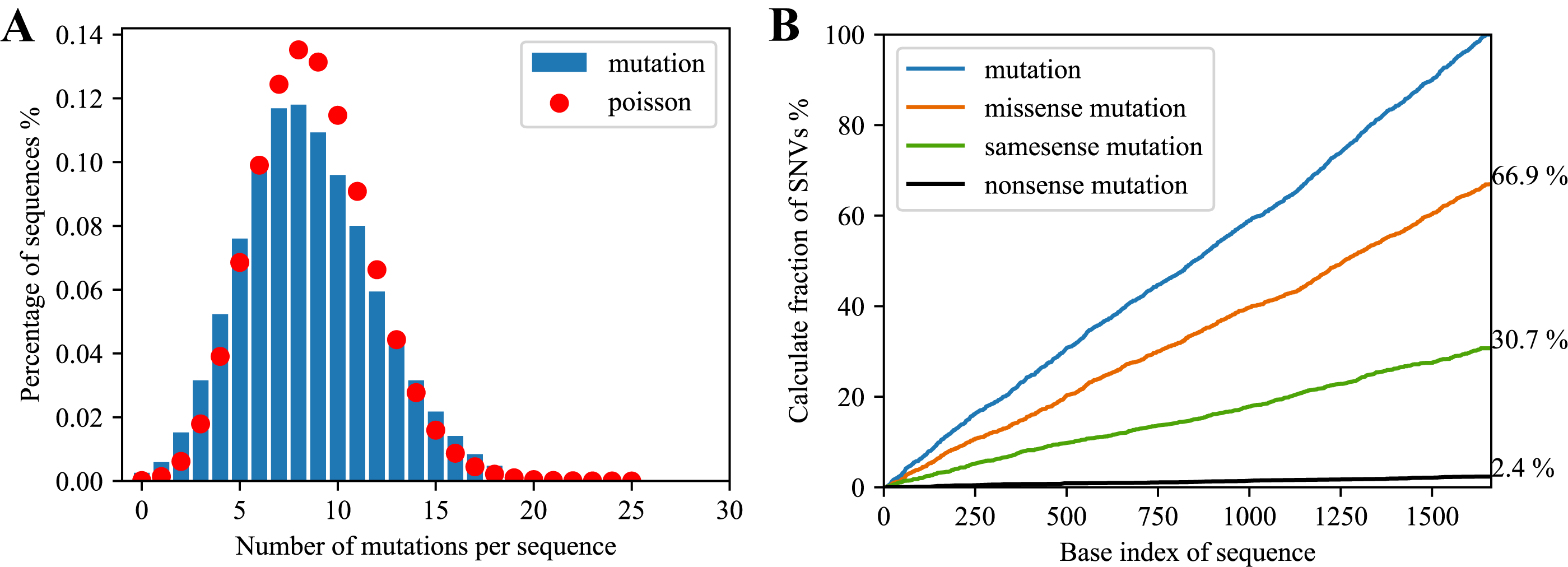

Supplement: Figure S1 — Overview of the random mutations in the HA gene of the of the mutant viral library. [file jvi.00151-25-s0001.tif]

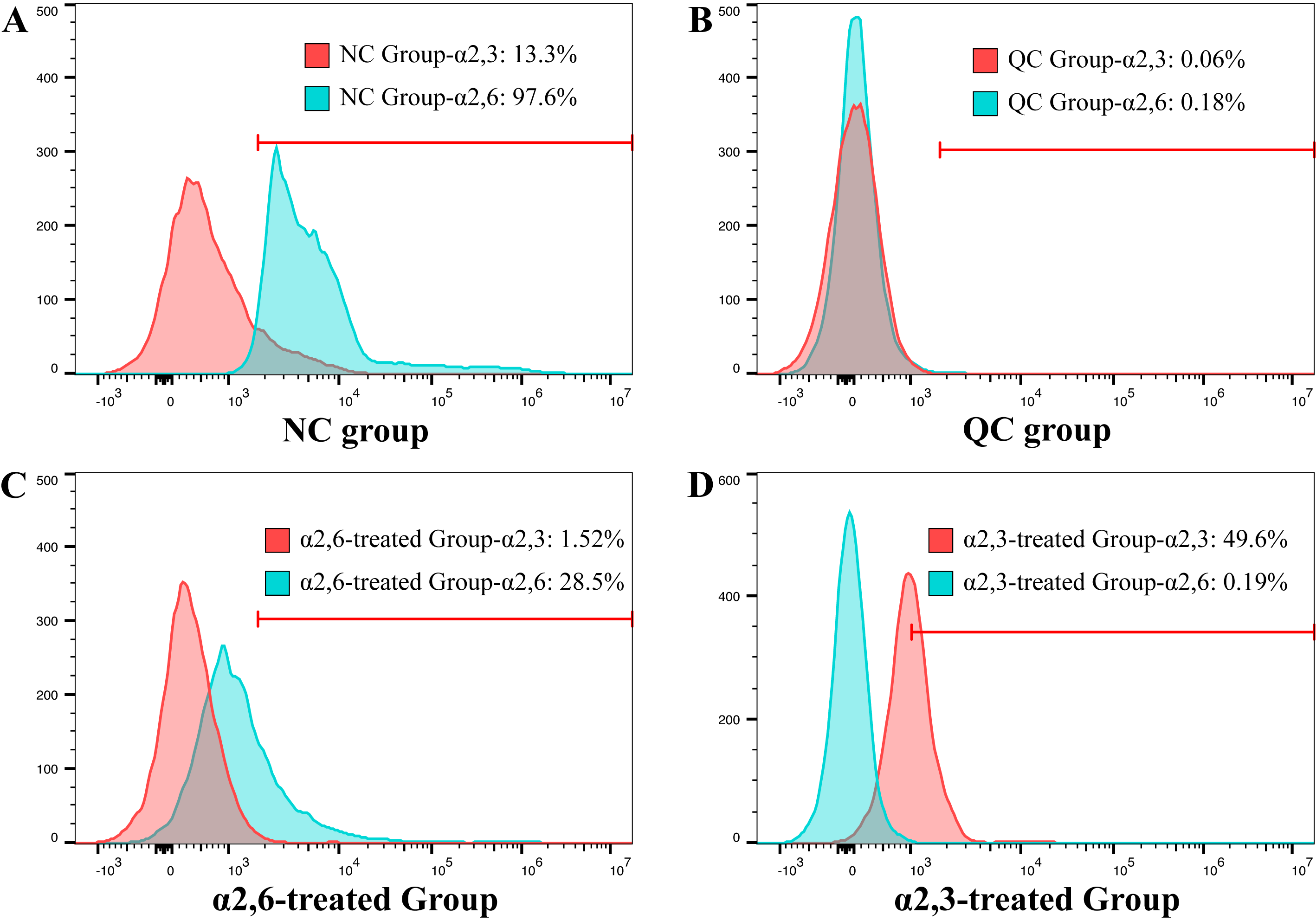

Supplement: Figure S2 — Binding affinity of MAL II or SNA to turkey red blood cells assessed through flow cytometry analysis. [file jvi.00151-25-s0002.tif]

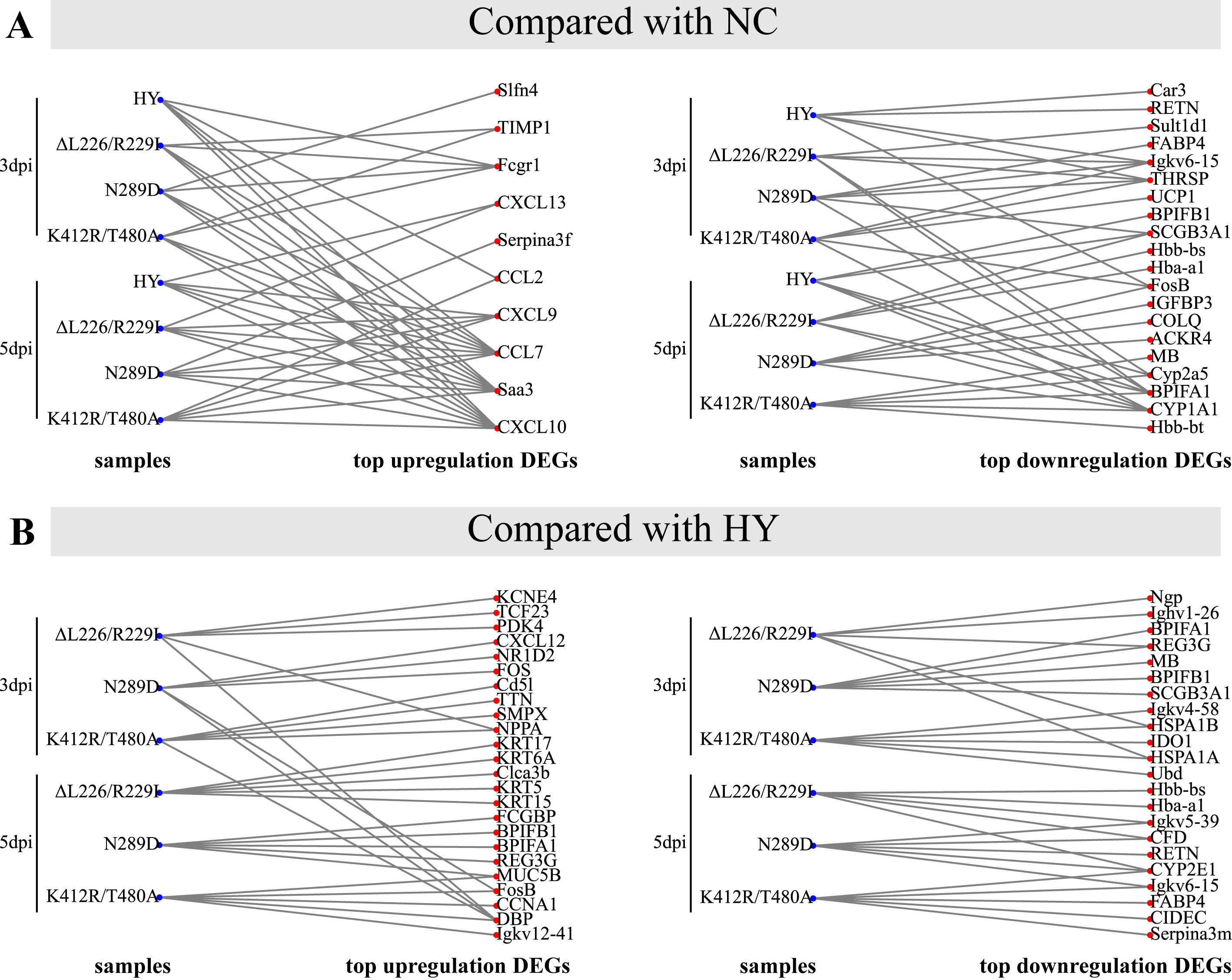

Supplement: Figure S3 — Link diagrams connecting the most differentially expressed genes to samples collected at various time points and from different mutant viral strains. [file jvi.00151-25-s0003.tif]

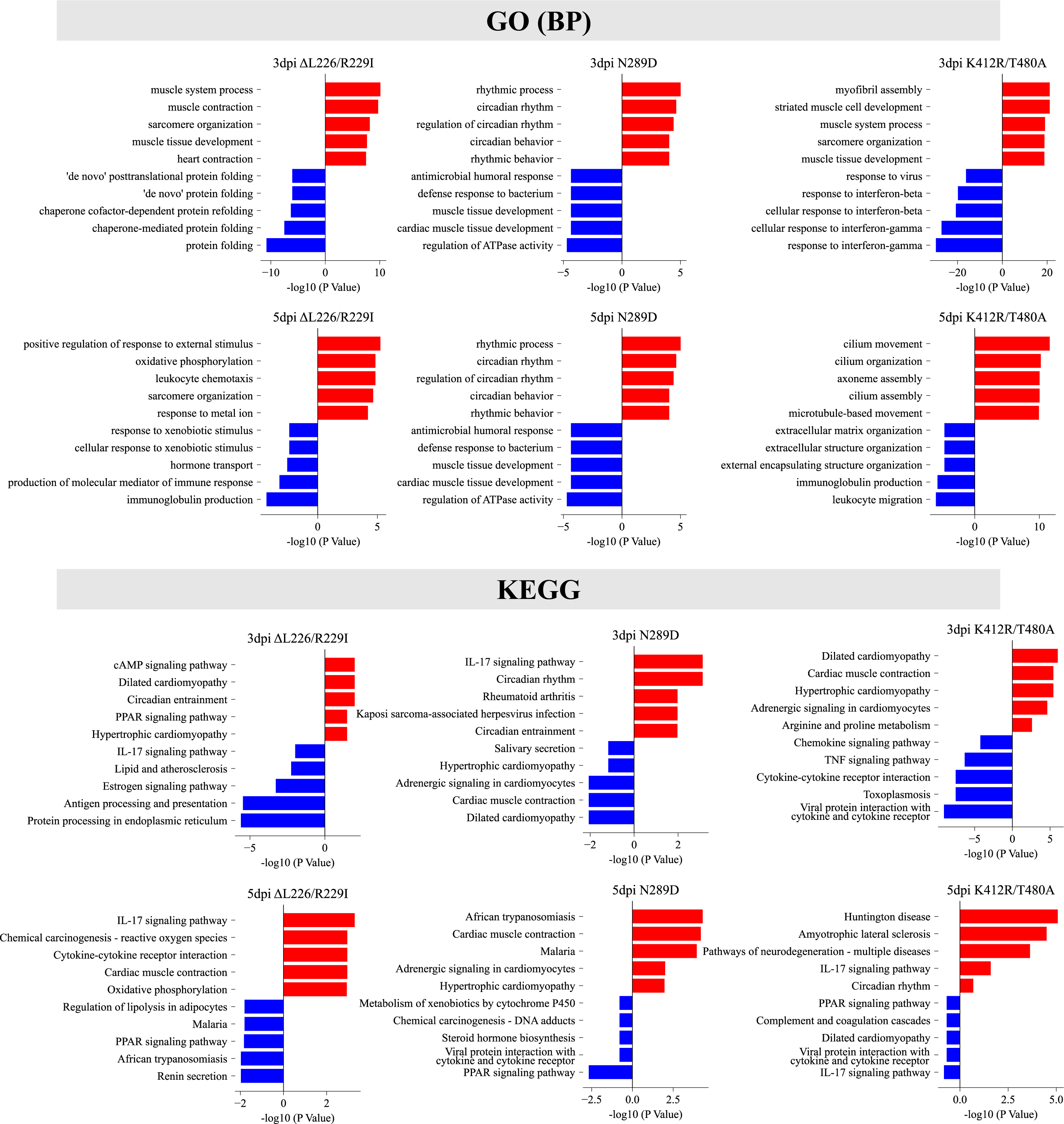

Supplement: Figure S4 — Bidirectional bar chart displaying the results of an enrichment analysis for DEGs in mice infected with various mutant HA viral strains at 3 and 5 dpi. [file jvi.00151-25-s0004.tif]
