# Supplementary material for: Adaptive selection of quasispecies during in vivo passaging in chickens, mice, and ferrets results in host-specific strains for the H9N2 avian influenza virus
Source: J Virol. 2025 May 8;99(6):e00151-25. doi: 10.1128/jvi.00151-25 (PMC12172485; doi:10.1128/jvi.00151-25)
Supplement: Supplemental material — Tables S1 to S3; legends for Fig. S1 to S4. [file jvi.00151-25-s0005.docx]

**Figure S1.** **Overview of the random mutations in the HA gene of the of the mutant viral library.** (A) The distribution of variant sequences with different numbers of mutations. On average, 8.7 mutations occurred per variant. The mutation frequency per variant approximately adheres to a Poisson distribution. (B) Accumulation of mutations in the nucleotide sequences of the HA gene. 66.9% of the mutations were missense mutations, 30.7% were synonymous mutations and 2.4% were nonsense mutations.

**Figure S2.** **Binding affinity of MAL II or SNA to turkey red blood cells (TRBCs) was assessed through flow cytometry analysis. The normal** TRBCs (NC group), de-sialylated TRBCs (QC group), Siaα2,6-TRBCs and Siaα2,3-TRBCs were stained with the sialic acid-specific lectin MAL II (preferentially recognizes Siaα2,3) or SNA (preferentially recognizes Siaα2,6) and characterized by flow cytometry.

**Figure S3:** Link diagrams connecting the most differential expressed genes (DEGs) to samples collected at various time points and from different mutant viral strains. The blue nodes represent the various samples, while the red nodes indicate the DEGs. A connection between a blue node and a red node signifies that the corresponding gene is classified as a the DEG in that specific sample. The left panel shows the upregulated DEGs, whereas the right panel displays the downregulated DEGs.

**Figure S4:** Bidirectional bar chart displaying the results of an enrichment analysis for DEGs in mice infected with various mutant HA viral strains at 3 and 5 dpi. The y-axis displays the enriched Gene Ontology (GO) Biological Process (BP) terms or Kyoto Encyclopedia of Genes and Genomes (KEGG) pathways. The length of the bars represents the significance of enrichment for the corresponding term/pathway, quantified as -log10(*P* value). Red bars indicate terms/pathways that are enriched among the upregulated genes, while the blue bars represent those enriched among the downregulated genes.

**Table S1:** Frequency of major substitutions in the HA gene after screening with Siaα2,6-TRBCs and Siaα2,3-TRBCs.

|  | Siaα2,6-TRBCs | Siaα2,3-TRBCs |
| --- | --- | --- |
| K412R | 94.6% | 3.5% |
| T480A | 94.7% | 2.5% |
| ΔL226 | 2.9% | 90.4% |
| R229I | 1.0% | 94.2% |

**Table S2**: Primers used to introduce mutations into the HA gene.

| Primer name | Primer sequence | Direction of the primer |
| --- | --- | --- |
| K412R-F | ACATGATCAATAATAGGGTTGATGATCAAAT | Forward |
| K412R-R | TTTGATCATCAACCCTATTATTGATCATGTT | Reverse |
| T480A-F | ATTGCATGGAGGCAATTCG | Forward |
| T480A-R | ATTCCGAATTGCCTCCATG | Reverse |
| ΔL226-F | CCCCTTGTCAACGGTATGGGAAGAAT | Forward |
| ΔL226-R | TTAATTCTTCCCATACCGTTGACAAGGG | Reverse |
| R229I-F | CGGTTTGATGGGAATCATTAATTATTATTGGT | Forward |
| R229I-R | ACCAATAATAATTAATGATTCCCATCAAACCG | Reverse |
| N289D-F | GGTGGCTTAGACACAACATTG | Forward |
| N289D-R | CAATGTTGTGTCTAAGCCACCT | Reverse |
| E439K-F | CAGAAAACACTCGATAAACATGACGC | Forward |
| E439K-R | CGTCATGTTTATCGAGTGTTTTCT | Reverse |
| L226M-F | TCAACGGTATGATGGGAAGAAT | Forward |
| L226M-R | ATTCTTCCCATCATACCGTTGAC | Reverse |
| M227V-F | CAACGGTTTGGTGGGAAGAA | Forward |
| M227V-R | AATTCTTCCCACCAAACCGTTGAC | Reverse |
| E106G-F | CCGGGAATGTAGGAAATCTAGAAG | Forward |
| E106G-R | CTTCTAGATTTCCTACATTCCCGGG | Reverse |
| T291A-F | GCTTAAACACAGCATTGCCCTTC | Forward |
| T291A-R | AAGGGCAATGCTGTGTTTAAG | Reverse |
| T291P-F | GCTTAAACACACCATTGCCCTTC | Forward |
| T291P-R | AAGGGCAATGGTGTGTTTAAG | Reverse |
| S203N-F | CACAACAACGAATGTGGCAAC | Forward |
| S203N-R | TGTTGCCACATTCGTTGTT | Reverse |

**Table S3:** Viral titers of 19 recombinant H9N2 viruses.

| Virus | log_10_ EID_50_/mL |
| --- | --- |
| HY | 9.08 |
| R229I | 8.20 |
| ΔL226 | 8.50 |
| ΔL226/R229I | 8.54 |
| K412R | 9.87 |
| K412R/T480A | 9.53 |
| T480A | 8.37 |
| E439K | 8.70 |
| N289D | 9.45 |
| M227V | 8.92 |
| L226M/M227V | 8.95 |
| L226M | 9.08 |
| L226M/R229I | 9.00 |
| S203N/L226M/M227V | 9.20 |
| L226M/M227V/T291A | 9.53 |
| T291A | 9.08 |
| T291P | 9.08 |
| S203V | 9.20 |
| E106G | 9.45 |
